# CT scan bilateral interstitial pneumonia caused by SARS-CoV 2

**DOI:** 10.11604/pamj.supp.2020.35.2.23114

**Published:** 2020-05-06

**Authors:** Danilo Coco, Silvana Leanza

**Affiliations:** 1 Department of General Surgery, Ospedali Riuniti Marche Nord, Pesaro, Italy; 2 Department of General Surgery, Carlo Urbani Hospital, Jesi, Ancona, Italy

**Keywords:** SARS-CoV 2, Bilateral Interstitial Pneumonia, X-ray, CT Scan

## Image in medicine

**Figure 1:**
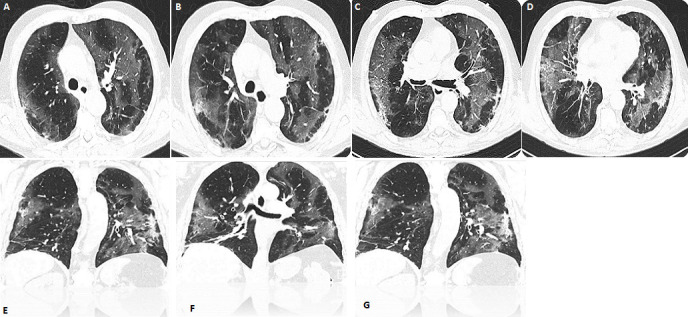
(A,B,C,D,E,F,G) CT Scan showed bilateral peripheral ground-glass opacities

SARS-CoV-2 is the coronavirus responsible for the COVID-19 pandemic of 2020. The mean incubation time is 5.1 days (95% CI: 4.5-5.8 days), with 97.5% of those who develop symptoms within 11.5 days (95% CI: 8.2-15.6 days). Mortality rates are currently unknown: from 0.25% to 10%. Currently, no vaccine is available. A 74-year-old man with a past medical history of LNH and CHT, presented to the Emergency Department in March after fever, cough, ageusia, anosmia at home for 20 days. He had had no recent travel outside of the state or internationally. Admission vital signs were normal. The results of routine laboratory parameters are shown: leucocytes 10,78x10^3^/mmc, lymphocytes 40%, D-DIMERO 1.739ng/mlFEU,PCR 20,54mg/dl, Pro-CALCITONIN 0,20ng/ml, ferritin 2.925,0ng/ml. Arterial Blood Gases pH 7,50,PCO2 27,0 mmHg, PO2 46,0 mmHg, PO2/FiO2 (P/F ratio) 219,0 mmHg. Nose and throat samples for SARS-CoV-2 PCR were positive. Examination of the lungs reveals murmure reduced. The test result returned positive. A CT chest showed bilateral peripheral ground-glass opacities (figure 1). He was treated with oxygen therapy, azithromicin 500mg/day, hydrossicloroquine 400mg/day and enoxaparin. Tocilizumab was not necessary. The patient improved and was discharged 15 day after.

